# Management of lung cancer patients during COVID-19 pandemic: dos, don’ts and don’t knows

**DOI:** 10.37349/etat.2022.00085

**Published:** 2022-06-10

**Authors:** Mariangela Torniai, Veronica Agostinelli, Luca Cantini, Carolina Liguori, Francesca Morgese, Silvia Rinaldi, Laura Scortichini, Rossana Berardi

**Affiliations:** 1 UOC Oncologia, Ospedale A. Murri, 63900 Fermo, Italy; 2 Clinica Oncologica, Università Politecnica delle Marche, AOU Ospedali Riuniti di Ancona, 60126 Ancona, Italy; University of Turin, Italy

**Keywords:** Coronavirus disease 2019, lung cancer, guidelines

## Abstract

**Aim:**

During the coronavirus disease 2019 (COVID-19) pandemic two needs have overlapped: on one hand continuing to provide the best care for patients with lung cancer and preventing the spread of the virus between patients and healthcare professionals on the other hand. Due to the pandemic’s unpredictable duration, physicians had to evaluate the risk/benefit ratio of anti-cancer therapeutic strategy to do the best for their patients and to protect patients themselves, as well as healthcare workers.

**Methods:**

Systematic literature research was performed with the aim to assess the available guidelines for the management of lung cancer patients during the COVID-19 pandemic. Thirteen potentially relevant articles were selected and recommendations have been divided into three main categories: dos, don’ts and don’t knows.

**Results:**

All guidelines and recommendations highlighted the relevance of being able to delay, if possible and based on risk stratification, and curative interventions. The selected recommendations should be considered adaptable and flexible because they might be contextualized on the basis of severe acute respiratory syndrome coronavirus 2 (SARS-CoV-2) infection prevalence and the availability of diagnostic-therapeutic resources.

**Conclusions:**

It remains of fundamental importance to discuss each diagnostic and therapeutic decision with the patient taking into account risks and benefits that might vary from case to case.

## Introduction

Coronavirus disease 2019 (COVID-19) is an infectious disease caused by severe acute respiratory syndrome coronavirus 2 (SARS-CoV-2), first identified in December 2019 in Wuhan, China. COVID-19 spread rapidly throughout China and worldwide until the pandemic was declared on 11th March 2020. On 18th June 2021, the total number of confirmed cases reached over 177 million with more than 3.8 million reported deaths [1].

Although approximately 81% of the cases of COVID-19 were classified as mild, 14% were severe and required hospitalization, and 5% were critical, with respiratory failure, septic shock, and/or multiple organ dysfunction or failure [2]. Since COVID-19 spreads primarily via droplets and no vaccine was available until January 2020, the only effective actions to prevent or delay community spread were containment, testing and isolation of cases, and social distancing. The COVID-19 pandemic has stressed the healthcare system and forced us to reallocate resources. Many clinical activities and elective surgeries were deferred in order to reduce patient traffic and avoid virus spread.

Cancer care has been adapted and reorganized, trying to minimize the patients’ risks. Patients with cancer may be at higher risk of morbidity and mortality related to COVID-19 than the general population because of coexisting chronic comorbidities, underlying malignancy, and systemic immunosuppressive states caused by antineoplastic therapy and cancer itself [3–5].

A particularly vulnerable group is represented by patients with lung cancer. This is due to their relatively older age at presentation, presence of baseline compromise pulmonary function, and other comorbidities. Furthermore, lung cancer patients often present with symptoms that overlap with COVID-19, including cough and dyspnea. Radiological manifestations of COVID-19-induced pneumonia include pleural effusion, extensive small lung nodules, irregular interlobular or septal thickening, and adenopathies, especially in the advanced phase. Computed tomography (CT) findings can overlap with those that are often found in patients with lung cancer upon disease progression or onset of concomitant pneumonia due to opportunistic infections [6].

The TERAVOLT study [7] retrospectively evaluated 200 lung cancer patients diagnosed with SARS-CoV-2 at the beginning of the COVID-10 pandemic reporting a lower admission to intensive care and higher mortality among patients with thoracic cancer, with an increased risk of death, especially in smokers, patients with comorbidities, older than 65 years and receiving treatment with chemotherapy alone.

Balancing the risk of infection with COVID-19, potentially life-threatening, with the consequences of delaying or not treating a highly life-threatening cancer is a current challenge. Today new strategies and priorities need to be identified in order to adapt the current well-defined standard guidelines for the management of lung cancer [8].

In this regard, this review focuses on features of COVID-19 infection in patients with lung cancer, aiming to improve their management.

## Materials and methods

**Figure 1.**
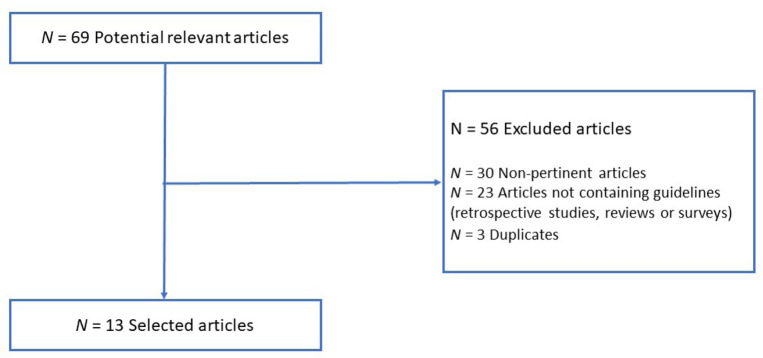
PRISMA

Systematic literature research was performed through the Pubmed database using the following keywords: “COVID-19”, “lung cancer”, “guidelines” and “recommendations”. All the specified keywords were combined using the “AND” operator. The selection was performed by searching for clinical practice guidelines published over the past five years (from 2017 to 2021), exclusively in the English language. This systematic review adheres to Preferred Reporting Items for Systematic Reviews and Meta-Analyses (PRISMA) guidelines [9]. After the analysis, we identified 13 potentially relevant articles. Fifty-six papers were excluded due to non-pertinent articles, duplicates, and articles not containing guidelines (retrospective studies, reviews, or surveys) (Figure 1). The analysis aimed to assess the available guidelines for the management of lung cancer patients in the COVID-19 era by examining and resuming recommendations into three main categories: dos, don’ts, and don’t knows.

## Results

During the COVID-19 outbreak, several Oncology Scientific Societies renewed recommendations or guidelines on the management of lung cancer patients, adapting diagnostic and therapeutic pathways to the new challenges imposed by the pandemic. Furthermore, new guidelines focusing on the management of lung cancer patients during the COVID-19 pandemia have become available.

### Dos

During the COVID-19 pandemic, two needs have overlapped: continuing to provide the best care for patients with lung cancer on one hand and preventing the spread of the virus between patients and healthcare professionals on the other hand. The recommendations included in this first category (do) have been divided among the following areas: screening, outpatients visit priorities and indication to radiological examinations, lung cancer diagnosis, management of localized disease, and stage III in patients with non-small cell lung cancer (NSCLC), follow up procedures, lung cancer radiotherapy, management of metastatic disease in NSCLC, small cell lung cancer (SCLC) patients’ management, palliative care and management of thoracic neuroendocrine neoplasms (NENs) (Table 1).

**Table 1.** The recommendation in lung cancer management

**Setting**	**Recommendation**
Lung cancer screening (LCS)	1- The screening might be delayed 2- Invasive procedures and surgery for patients with intermediate-risk nodules should be deferred and a PET scan and/or nonsurgical biopsy should be preferred 3- For high-risk nodules proceed with an empiric treatment decision without further diagnostic testing
Lung cancer outpatients management	1- Temperature check and questionnaire for detecting symptoms of COVID-19 for patients accessing to the hospital 2- Patients with signs and symptoms highly suspected of lung cancer should be managed within standard pathways, avoiding delays, while radiological investigations can be delayed in other cases 3- Visits may be converted into telemedicine visits during follow up and in patients on active treatment with oral drugs 4- Psychosocial support should be assured and, when possible, converted to telemedicine
Lung cancer diagnosis	1- Diagnostic imaging (e.g., CT, PET) should be scheduled on the same day while preoperative full lung function testing should be avoided 2- Bronchoscopy should be avoided if not necessary and percutaneous procedures should be preferred 3- Mediastinoscopy should be performed at the same time as surgery 4- Virtual modality for the multidisciplinary meeting should be preferred
Management of localized disease in NSCLC patients	1- All patients with a high suspicion or histological diagnosis of lung cancer should undergo surgical resection to avoid delays 2- Patients should be referred to the nearest thoracic surgery center, except for patients with locally advanced lung cancer that should be addressed to high-volume thoracic surgery centers 3- Minimally invasive access should be considered as the first option whenever possible 4- A telephone triage is strongly recommended as well as a nasopharyngeal swab testing for SARS-CoV-2 in the previous 48 h 5- In non-urgent patients affected by COVID-19 infection, surgery should be deferred for at least 14 days and until infection resolution is demonstrated with the repeated negative nasopharyngeal swab test 6- The indication for adjuvant chemotherapy should be strongly considered in young patients (< 65 years old) with resected pT3/T4 tumors or in case of pN2 disease or the presence of negative prognostic features 7- The use of G-CSF a priori in patients treated with platinum-based chemotherapy in neoadjuvant and adjuvant settings could be considered
Management of stage III NSCLC	1- Therapeutic strategies should be set up within a multidisciplinary team 2- For patients who are candidates for perioperative chemotherapy, a neoadjuvant approach should be preferred 3- Concomitant or sequential chemoradiotherapy and possible maintenance with durvalumab (repeated every 4 weeks instead of 2 weeks) should be ensured without delay
Follow-up	1- For stage I NSCLC follow-up imaging and visits should be postponed 2- For stage II or III NSCLC treated with a curative intent with no new symptoms, follow-up imaging can be postponed while visits and clinical check-ups should be maintained preferring telemedicine 3- For stage II or III NSCLC treated with palliative intent with no new symptoms follow-up imaging and visits can be postponed up to 6 months; however, when feasible, telemedicine follow-up visits are recommended almost every 3 months
Lung cancer radiotherapy	1- Radiotherapy treatment should not be delayed, especially when is part of a multimodal approach for curative purposes or represent a palliative treatment while PORT in patients with completed resected NSCLC and PCI in patients with SCLC may be postponed 2- Hypo-fractionating should not replace usual fractionation except for palliative treatment 3- Operable patient with stage I NSCLC should receive SBRT when access to surgery is not available due to surgical capacity issues 4- COVID-19 positive patients should delay radiotherapy until the test for COVID-19 is negative
Management of metastatic disease in NSCLC	1- First-line and second-line cancer treatments in symptomatic patients should not be delayed considering the use of G-CSF 2- At diagnosis, the biological characterization of the disease remains fundamental to direct the correct therapy 3- The schedule of ICIs should be modified to limit clinical visits and in patients treated with ICIs for more than 12–18 months, the delay or omission of some cycles might be considered to evaluate the possibility to stop ICIs after two years 4- In oncogene-addicted diseases, treatment with TKI must be continued preferring telemedicine visits 5- In patients undergoing chemotherapy treatment, it is preferable to switch from intravenous to oral formulations 6- For antiresorptive bone-protective therapy (zoledronic acid, denosumab) a temporary withdrawal should be considered 7- Oncological treatments with a low probability of efficacy should be carefully evaluated and discussed on a case-by-case basis evaluating the risk-benefit ratio 8- Transfusion of blood or platelets should be avoided using dose reduction or delay in chemotherapy administration 9- Imaging assessment: slots for imaging within 6 months from treatment start or in case of suspected progression disease should be performed
SCLC patients’ management	1- Treatment of SCLC remains a priority 2- Oral etoposide can be considered instead of intravenous etoposide to reduce time in hospital 3- In first-line treatment, the addition of the maintenance treatment with an ICI (atezolizumab or durvalumab) might be omitted due to the imitated improvement in OS 4- G-CSF support is strongly recommended for patients with a high or medium risk of febrile neutropenia 5- PCI should be postponed in patients with limited-stage and replaced by close follow-up in patients with the extensive disease while consolidation thoracic irradiation should be avoided 6- The start of a second line should be evaluated case by case with a risk-benefit balance
Palliative care	1- Patients with recurrent pleural effusions should receive evacuative thoracentesis or thoracoscopy with talc poudrage, when indicated 2- In patients with severe dyspnea for central or endobronchial lung cancer, mechanical dilatation, laser to remove obstructions, and airway stenting are recommended 3- Home palliative care should be preferred, whenever possible, through virtual contact with patients, their family members, and home care staff
Management of thoracic NENs	1- Multidisciplinary management remains fundamental in using virtual platforms 2- Home treatments and local pharmacies for drugs supply should be preferred for performing locally radiological or laboratory investigations 3- In patients with active COVID-19 infection, anticancer treatment should be delayed until swab negativization 4- Curative surgery should take precedence over metastasis resection and debulking procedures 5- Palliative radiotherapy should be delayed in asymptomatic patients 6- A cycle delay or omission of a cycle should be individually considered in patients treated with PRRT

PET: positron emission tomography; G-CSF: granulocyte-colony stimulating factor; PORT: post-operative radiotherapy; PCI: prophylactic cranial irradiation; SBRT: stereotactic body radiation therapy; ICIs: immune checkpoint inhibitors; TKI: tyrosine kinase inhibitors; PRRT: peptide radionuclide receptor therapy; OS: overall survival

#### LCS

Despite screening for lung cancer has been hotly debated, currently, national and international guidelines recommend annual low-dose chest CT for high-risk individuals. The COVID-19 pandemic reduced healthcare resources slowing down screening programs and non-urgent procedures. A panel of experts developed a consensus statement to establish specific recommendations for LCS during the COVID-19 pandemic [10]. In particular:

1- The beginning of screening might be delayed.
2- The screening for eligible smokers’ required annual chest CT scan during the COVID-19 pandemic should be delayed.
3- The surveillance chest CT scan for evaluation of a screening-detected lung nodule or pure ground-glass nodule, should be delayed for approximately 3 months to 6 months.
4- For the management of patients with intermediate-risk nodules it is acceptable to defer invasive procedures and surgery and to oversee the patient with a PET scan and/or nonsurgical biopsy to evaluate the nature of the nodule and the necessity of surgery.
5- For the management of patients with high-risk nodules it is reasonable to proceed with an empiric treatment decision (surgery or stereotactic radiotherapy) without further diagnostic testing to minimize exposure to the healthcare environment.

#### Lung cancer outpatient visit priorities and an indication of radiological examinations

During the pandemic, outpatient visits represent a risk of virus spread. Therefore, outpatient cancer services should be reinforced and reorganized. A European Society for Medical Oncology (ESMO) panel of experts defined some recommendations [11]:

1- Upon access to the hospital, the patient should undergo a temperature check and a rapid questionnaire for detecting possible symptoms of COVID-19.
2- Patients with signs and symptoms highly suspected of lung cancer should be managed within standard pathways, avoiding delays.
3- Follow-up visits may be converted into telemedicine visits. Radiological investigation can be delayed in low-intermediate risk of relapse and asymptomatic patients.
4- Patients on active treatment with oral drugs, may benefit from telemedicine and blood tests should perform at home.
5- Psychosocial support should be assured and, when possible, converted to telemedicine.
6- Discussing with oncologists, radiologists should postpone and reschedule the non-urgent diagnostic or image-guided procedure.

#### Lung cancer diagnosis

During the pandemic, the diagnostic phase is crucial to appropriately select patients suitable for surgery. Experts defined some recommendations [12]:

1- All patients with suspected lung malignancy could have priority access to diagnostic imaging (e.g., CT, PET); however, staging exams should be concentrated on the same day.
2- PET has a fundamental role in the differential diagnosis between benign lesions and malignant neoplasms. During the pandemic, PET should be performed on all patients with suspected lung cancer suitable for surgery.
3- During the pandemic, bronchoscopy, when performed for completion of staging, should be avoided as it represents a procedure with high-risk infection for other patients and medical staff. However, it remains necessary to plan surgical strategy when a bronchial or carinal reconstruction should be performed.
4- For diagnosis and staging, percutaneous procedures should be preferred, possibly in outpatient or day hospital facility.
5- Diagnostic algorithms, for managing the solitary pulmonary nodule, should be performed to identify the risk of malignancy and to guide the physicians towards appropriate diagnostic tests, when indicated. Several diagnostic algorithms have been developed, combining clinical risk factors with radiological signs. The most important factors to take into account are age, smoking habit, hemoptysis, history of malignancy, nodule diameter, location, edge characteristics, growth rate, cavity wall thickness, calcification, and contrast enhancement on CT scan > 15 HU and PET scan results. Stratifying patients in risk class, during the pandemic the followed recommendations have been established:
  √ Elective surgery should be postponed for 3 months for lung nodules with a probability of benignity > 70%.
  √ Cytological/histological diagnostic procedure should be considered in patients with lesions defined by a high risk of malignancy.
  √ Surgery should be performed when the diagnosis of lung cancer is reached.
  √ Follow-up studies performed within 1 month are recommended when the diagnosis of lung cancer is not reached.
6- Ground-glass opacity (GGO) is defined purely with a solid component, according to the radiological aspect. Several causes can determine GGO including SARS-CoV-2 and lepidic lung adenocarcinoma, and differential diagnosis is complex during the COVID-19 outbreak the experts suggest:
  √ CT scan after 3 months for pure GGO or mixed GGO lesion with solid component < 50%.
  √ Surgical resection without further characterization in GGO lesion with predominant solid component > 50%.

Furthermore, a lung function examination should be performed before surgery. However, spirometry could increase the risk of COVID-19 contagion for patients and medical staff. Therefore, several respiratory societies suggest avoiding airway challenge testing, preferring analysis of blood gases and spot-check oximetry. With a correct cleaning of equipment and surrounding areas after each patient, spirometry is still recommended in patients with previous respiratory failure, unfit patients, or in patients who are a candidate for extended surgical resection. Adequate personal protective equipment (PPE) associated with appropriate precautions should be maintained to lower the risk of infection due to aerosol-generating procedures [13].

Mediastinoscopy could be performed at the same time as surgery, in order to reduce hospital admissions [12].

Phone COVID-19 anamnestic questionnaire and triage including patient’s evaluation with body temperature and signs and symptoms collection should be performed before hospitalization [13].

It is preferable to plan multiple procedures in single hospital access (i.e. biopsy, lab exams, visit, pleural interventions) and all treatment plans for lung cancer patients need to be discussed in a multidisciplinary context since it improves patients’ outcomes. The difference may be the way of meeting (e.g., videoconferencing) [11].

#### Management of localized disease in NSCLC patients

During the pandemic, it is fundamental referring lung cancer patients to higher-volume surgical centers allowing them to perform also complex lung cancer resections and prevent and manage possible complications. Thoracic surgery represents a high risk of virus spread, exposing healthcare workers to a high risk of infection. In fact, several aerosol-generating procedures are necessary for thoracic surgery patients with lung cancers such as endoscopic procedures, endotracheal intubation, and complex ventilation procedures. A European high-volume referral center suggests that medical institutions should adopt separated therapeutic pathways for patients who are suspected of being positive or have a diagnosis of COVID-19 infection, from non-infected patients. During the COVID-19 outbreak the experts suggest [13, 14]:

1- All patients with a high suspicion or histological diagnosis of lung cancer should undergo surgical resection.
2- To minimize interregional displacements, patients should be referred to the nearest thoracic surgery center, except for patients with locally advanced lung cancer that should be addressed to high-volume thoracic surgery centers.
3- All patients candidate for multimodal treatment, including surgery, should receive fast-track surgical procedure.
4- Surgery delay should be avoided when hospital resources are still intact as it can be detrimental to patients’ survival.
5- During the pandemic it is crucial to reduce the length of stay; minimally invasive access guarantee equivalent oncologic outcomes and it should be considered as the first option whenever possible.
6- A correct telephone triage the day before and in person at the time of admission, to identify suspected cases of acute infection or direct contact with sick subjects is strongly recommended.
7- Nasopharyngeal swab testing for SARS-CoV-2 in the previous 48 h is strongly recommended.
8- In non-urgent patients affected by COVID-19 infection, surgery should be deferred for at least 14 days and until infection resolution is demonstrated with the repeated negative nasopharyngeal swab test.

Adjuvant chemotherapy with cisplatin and vinorelbine represents the standard of care in resected stages I to III of NSCLC, with an absolute 5-year survival improvement of about 5%. Due to immunosuppressive risk, during the COVID pandemic, the role of adjuvant chemotherapy should be reconsidered, taking into account risk factors of tumor relapse and comorbidities. During the COVID-19 outbreak the experts suggest [8, 11]:

1- The indication should be strongly considered in young patients (< 65 years old) with resected T3/T4 tumors or in case of pN2 disease or the presence of negative prognostic features (e.g., lymphovascular infiltration, pathological lymph node invasion).
2- The use of G-CSF should be considered to reduce neutropenia and its related risk of hospitalization.

#### Management of stage III NSCLC

The management of stage III disease is complex and requires multimodal treatment involving a surgeon, radiotherapist, and oncologist. During the COVID-19 outbreak the experts suggest [11, 12, 15]:

1- Therapeutic strategies should be set up within a multidisciplinary team. Virtual tumor board meetings should be considered.
2- For patients who are candidates for perioperative chemotherapy, a neoadjuvant approach should be preferred (recommended schemes with less risk of hospitalization and number of visits).
3- Due to the significant curative aim, the treatment should receive high priority. Concomitant or sequential chemoradiotherapy and possible maintenance with durvalumab should be ensured without delay. However, a schedule containing cisplatin/pemetrexed instead of cisplatin/etoposide, or weekly carboplatin/paclitaxel should be considered in non-squamous NSCLC to limit hospital admissions. Furthermore, durvalumab infusion can be repeated every 4 weeks instead of 2 weeks, where the National Regulatory Agency allows it.

#### Follow-up

During the COVID-19 pandemic the experts suggest [12, 14]:

1- For stage I NSCLC asymptomatic patients, follow-up imaging and visits should be postponed.
2- For stage II or III NSCLC, who received a curative treatment (surgery or chemoradiotherapy) completed over more than 1 year and with no new symptoms, follow-up imaging can be postponed up to 1 year. When feasible, visits and clinical check-ups should be maintained.
3- For stage II or III NSCLC, who received a curative treatment (surgery or chemoradiotherapy) completed less than 1 year and with no new symptoms, follow-up imaging and visits can be postponed up to 6 months.
4- During the follow-up period, telemedicine should be preferred to clinic visits.
5- For patients with NSCLC in stages II and III, treated with palliative intent and without evidence of disease activity, follow-up imaging and visits can be postponed up to 6 months. However, when feasible, telemedicine follow-up visits are recommended to check clinical status almost every 3 months.
6- For asymptomatic patients with SCLC in stages I and III, who received treatment with curative intent completed less than 2 years, follow-up imaging and visits can be postponed up to 6 months. For patients, who completed the treatment over more than 2 years, follow-up imaging and visits can be postponed up to 1 year. However, telemedicine follow-up visits are recommended every 3 months.

#### Lung cancer radiotherapy

During the COVID-19 pandemic, patients’ travel should be minimized to reduce the exposure of patients, family members, and health workers to SARS-CoV-2. A panel of experts belonging to the European Society for Radiotherapy and Oncology (ESTRO) and the American Society for Radiation Oncology (ASTRO) developed these recommendations [16]:

1- Timing: radiotherapy treatment should not be delayed, especially when it is part of multimodal treatment for curative purposes [NSCLC stage III and limited disease (LD)-SCLC] or palliative treatments. On a case-by-case basis, treatments such as PORT in patients with completed resected NSCLC and PCI in patients with SCLC may be postponed. Greater discrepancy emerged in the recommendation of curative radiotherapy in stage I of NSCLC. The experts conclude that it should be evaluated on a case-by-case basis taking into account the tumor growth rate, the share of ground glass *vs.* solid component, performance status, and patients’ will.
2- Schedule: a large consensus established that hypo-fractionating should not replace usual fractionation, except for palliative treatment.
3- Operable patients with stage I NSCLC should receive SBRT when access to surgery is not available due to surgical capacity issues.
4- COVID-19 positive patients should delay the beginning or interrupt radiotherapy until the test for COVID-19 is negative.

#### Management of metastatic disease

Treatment of patients with metastatic NSCLC aims to improve quality of life and survival. Treatment approaches are evidence-based, and, depending on the biological characteristics of the disease, it involves chemotherapy, immunotherapy, TKI, and different combinations. The COVID-19 pandemic represents a high risk of death for patients with lung cancer, however, the postponement of the treatments could exceed this death risk linked to SARS-CoV-2, therefore, the experts suggest [8, 11, 12, 15, 17, 18]:

1- First-line and second-line cancer treatments in symptomatic patients should not be delayed. At diagnosis, the biological characterization of the disease remains fundamental to directing the correct therapy.
2- G-CSF use should be considered in schedule with a risk of febrile neutropenia > 10% in order to reduce the risk of neutropenic sepsis and then the number of hospital admissions.
3- The schedule of ICIs should be modified in order to limit clinical visits, in particular using 4-weekly nivolumab 480 mg instead of the standard 2-weekly and 6-weekly pembrolizumab 400 mg instead of 3-weekly, when appropriate and allowed by National Regulatory Agency.
4- In patients treated with ICIs for more than 12–18 months, the delay or omission of some cycles may be considered. Furthermore, given the lack of data on the optimal duration and benefit of continuing immunotherapy after 24 months in responding patients, discontinuation of treatment after two years from the beginning could be evaluated.
5- In oncogene-addicted diseases, given the fundamental role of TKI and the risk of flare-up upon their discontinuation, treatment must be continued. However, telemedicine controls, blood samples were taken at home or in near-home laboratories, and drug delivery services should be considered to reduce hospital visits.
6- In patients undergoing chemotherapy treatment, it is preferable to switch from intravenous formulations to oral formulations (when available, e.g., vinorelbine, etoposide) with possible home delivery services to reduce hospital visits.
7- For antiresorptive bone-protective therapy (zoledronic acid, denosumab) a temporary withdrawal can be considered or administered at home whenever possible.
8- Oncological treatments with a low probability of efficacy should be carefully evaluated and discussed on a case-by-case basis by evaluating the risk-benefit ratio.
9- Transfusion of blood or platelets should be avoided using dose reduction or delay in chemotherapy administration. Furthermore, for the treatment of anemia secondary to chemotherapy darbepoetin alfa 3 weekly should be preferred and blood transfusions avoided.
10- Imaging assessment: during the pandemic, imaging devices, such as CT, are often used for patients with COVID. Patients receiving systemic therapies should be monitored and reviewed with alternative methods. However, slots for imaging within 6 months from treatment start or in case of suspected progression of disease should be available.
11- A telephone screening of patients should be performed the day before their visit to identify COVID-19-associated symptoms or positive contacts in the last 14 days. This questioning should be repeated upon their arrival at the clinic.
12- Health care staff, patients, and companions must use adequate PPE.
13- It is important to remember that cancer patients can present fever for many reasons (neoplastic fever, febrile neutropenia, pneumonia, sepsis), and the differential diagnosis with COVID-19 infection is essential for correct and timely therapy. Patients and their close contacts, who have suspected or confirmed COVID-19 infection, should respect the isolation, and should inform the referral cancer center of any quarantine requirements.
14- Enrollment in clinical trials represents an opportunity for patients and must be maintained. However, during the COVID-19 emergency, there could be deviations or violations of the protocol for limitation of national and international travels, therefore clinicians should contact the medical monitor or sponsor.

#### SCLC patients’ management

SCLC is an extremely aggressive disease with rapid growth and metastatic dissemination. The treatment of these forms must therefore be timely. During the pandemic experts advise [11, 12, 15]:

1- Treatment of SCLC remains a priority in patients eligible to receive first-line chemotherapy with or without ICIs for metastatic disease, and in patients with LD who are candidates for chemoradiotherapy treatments.
2- Oral etoposide can be considered instead of intravenous etoposide in order to reduce time in hospital.
3- In first-line treatment, the addition of the maintenance treatment with an ICI (atezolizumab or durvalumab) can be omitted due to the imitated improvement in OS.
4- G-CSF support is strongly recommended for patients with a high or medium risk of febrile neutropenia.
5- PCI should be postponed in patients with limited-stage and replaced by close follow-up in patients with extensive disease.
6- Consolidation of thoracic irradiation should be avoided.
7- The start of a possible second line should be evaluated case by case with a risk-benefit balance.

#### Palliative care

During the COVID-19 pandemic, patients with stage IV lung cancer requiring palliative care should be treated without delay. During the COVID-19 outbreak the experts suggest [12, 13]:

1- Due to significant quality-of-life improvement and the favorable benefit/risk ratio, patients with recurrent pleural effusions should receive evacuative thoracentesis or thoracoscopy with talc poudrage, when indicated.
2- In patients with severe dyspnea for central or endobronchial lung cancer mechanical dilatation or laser to remove obstructions and airway stenting are recommended.
3- Home palliative care should be preferred when possible.
4- Virtual contact with patients for best supportive care, their family members, and home care staff should be foreseen and provided to improve patients’ therapy when necessary and any psychological support.

#### Management of thoracic NENs

The management of patients affected by NENs requires in most cases a multidisciplinary approach. The COVID-19 pandemic creates a health crisis with a lack of healthcare personnel and cure availability. Furthermore, cancer patients presented a higher risk of morbidity and mortality related to COVID-19 infection due to clinical conditions and immunosuppressive treatments. Therefore, during the COVID-19 pandemic, it was required to de-escalate care to minimize the risk of exposure and most departments delayed the initiation of clinical trials to reduce visits. That situation produced repercussions on the research and development of new therapeutic strategies. The effect of treatment delays is unclear. A panel of experts defined revised strategies to assure correct and prompt treatments for NENs patients [8, 19]:

1- Multidisciplinary management is fundamental: virtual platforms or phones should be used for maintaining a multidisciplinary approach to care.
2- During the pandemic it is essential to inform and educate the patient about new ways of care and assistance: virtual care, reducing unnecessary visits when it is possible preferring home treatments, and local pharmacies for drugs supply.
3- Radiological or laboratory investigations should be postponed or performed locally when possible.
4- In patients with active COVID-19 infection, anticancer treatment should be delayed for a minimum of 14 days and/or until all symptoms resolution. Furthermore, treatments administered only in oncology should be deferred until the virus is no longer present (e.g., at least a negative COVID-19 test).
5- Due to reduced surgical activity, curative surgery should take precedence over metastasis resection and debulking procedures.
6- Palliative or curative radiotherapy should be delayed in asymptomatic patients. A single fraction is preferable in palliative treatment.
7- For carcinoids, treatment with somatostatin analogues (SSAs) should be maintained in functionally or symptomatic neoplasms. Home administration should be preferred. For asymptomatic patients with low-grade, slow-growing neoplasms, SSAs administration can be delayed.
8- Patients treated with everolimus should continue the treatment.
9- Cancer patients treated with PRRT during the COVID-19 pandemic didn’t show a shown increased susceptibility to risk of viral infections. However, a cycle delay, omission of a cycle, or extension of the interval between treatments should be individually considered, due to immunosuppressive risk.
10- Routinely withholding chemotherapy is not recommended. However, for the same efficacy, combinations with less need for intravenous fluids, such as carboplatin instead of cisplatin should be preferred and prophylactic growth factors should be considered for high-risk chemotherapy regimens.
11- Due to a lack of data supporting adjuvant therapy, patients undergoing radical surgery, in particular, those with favorable histologic features (i.e. negative margins, low Ki67) should not receive adjuvant therapy.

### Don’ts

Regarding the diagnostic process several authors, to limit disseminating droplets procedures suggested not performing diagnostic and staging bronchoscopy [12, 13], endobronchial ultrasound (EBUS), and echoendoscopy [12] favoring percutaneous procedures [8].

Focusing on lung cancer radiotherapy, ESTRO and ASTRO published their recommendations to lead practitioners’ decisions during the pandemic. Aiming at not compromising patients’ prognosis, they stated that in the early phase of the COVID-19 pandemic curative treatment for stage III NSCLC and limited stage LS SCLC must not be delayed. Moreover, it was agreed not to modify radiotherapy practice to more hypo-fractionated regimens, except when aimed at palliation and in early-stage NSCLC. According to current guidelines, almost all authors decided not to change multi-modality treatment and not to adopt induction strategies to delay radiotherapy in mutation-positive [epidermal growth factor receptor (EGFR) mutations or anaplastic lymphoma kinase (ALK) traslocation] lung cancer patients [16]. In managing locally advanced disease, concomitant radio-chemotherapy should not be deferred in patients who received multidisciplinary indication, as well as palliative irradiation treatment in life-threatening conditions (spinal cord compression, superior vena cava obstruction, symptomatic bone metastases, bleeding, severe dyspnea) must not be delayed or negated [11, 16]. Contrary, radiotherapy treatment in patients without nodal involvement post neoadjuvant treatment (ypN0), consolidation radiotherapy, as well as PCI in SCLC patients should not be proposed [8, 11, 12].

To minimize cancer patients’ and healthcare providers’ risks, some authors suggested relying on suspected radiological characteristics (for example, solid or predominantly solid lesions, larger than 2 cm), and avoiding further diagnostic exams before proceeding with surgical removal [10, 12]. Conversely, Fiorelli and colleagues [13] stated that is inappropriate to delay surgical treatment for lesions highly suggestive to be malignant even in the primary stages, suggesting performing PET to identify patients that should undergo surgery immediately, as also stated by the ESMO [11, 13]. In PET-negative lesions highly suspected to be lung cancer, all procedures to clearly identify the nature of a lesion must be used. To reduce the length of hospital stay and preserve appropriate and successful surgical pathways, thoracic surgeons suggested not to proceed with preoperative full lung function testing, narrowing spirometry to unfit patients or patients with anamnestic respiratory failure history, and to situations where a major surgical resection is required [13]. Contrary, the Sociedad Mexicana de Oncología highlighted that if spirometry results are adequate, no other evaluations are needed [14].

The British Thoracic Society (BTS) and the French Haut Conseil de la Santé Publique (HCSP) confirmed the indication to limit cancer patients’ hospital admissions. In particular, they agreed to not administer maintenance therapy in metastatic or not chemoradiotherapy eligible SCLC patients, due to the limited advance in OS, as well as pemetrexed in malignant pleural mesothelioma [15, 17]. Baldotto et al. [12] suggested that patients candidates for maintenance chemotherapy and immunotherapy should continue only anti-programmed cell death 1 (PD1) treatments, with an adequate dosage (pembrolizumab 400 mg i.v. every 6 weeks).

Additionally, to limit second-line therapy options, it is advisable to exclude cyclophosphamide/doxorubicin/vincristine in SCLC to avoid hospitalization. In elderly patients with NSCLC and Eastern Cooperative Oncology Group Performance Status (PS ECOG) ≥ 2 or remarkable comorbidity, authors considered the possibility to avoid adjuvant chemotherapy or discontinuing it maybe after 3 cycles, according to the limited benefit. Moreover, to limit the consumption of resources, they encouraged clinicians to start denosumab without a dentist consult and to avoid blood transfusions in palliative intent, favoring dose reduction or chemotherapy interruption [15]. Focusing on rare tumors, Rodriguez-Freixinos et al. [19] in their paper aimed to list indications in managing NENs patients during the COVID-19 epidemic, involving a multidisciplinary panel of 14 specialists. In the staging process, the authors recognized PET as not mandatory. In choosing treatments, they recommended not to prefer targeted therapies (sunitinib and everolimus), given their common related toxicities (as immune-suppression), and to pick up other treatments, such as PRRT. If targeted therapies are mandatory, they suggest examining to stop treatment or adopting a dose reduction, in case of prolonged stability disease. Finally, they propose that interferon α must be used with caution, due to flu-like symptoms that can be misread.

Moreover, giving necessary restrictions in i.v. fluid administration in pre-acute respiratory distress syndrome in COVID-19 disease, regarding platinum salts they decide on carboplatin. Finally, in COVID-19-positive NEN patients, they suggested not interrupting SSAs in symptomatic secretory NENs [19]. Don’ts are reassumed in Table 1.

### Don’t knows

In the context of routinely used systemic oncologic therapies, data were insufficient to estimate the relative risk of COVID-19 infection and related consequences. Oncologists played out a lot of approaches to guarantee to maintain patient care, but at the same time trying to avoid COVID-19 infections. Nowadays there are still questionable strategies, reassumed in Table 1.

To minimize neutropenia and infection related to chemotherapy-induced immunosuppression, risk of hospitalization, and risk of related nosocomial SARS-CoV-2 acquisition, the use of G-CSF could be considered a priori in patients treated with platinum-based chemotherapy in the neoadjuvant and adjuvant setting and in patients with metastatic NSCLC if the risk of neutropenia is above 10% [12]. It is important to underline however this strategy could not affect the specific risk of COVID-19 infection [11].

Nevertheless, some authors considered a dose reduction of chemotherapy could be an alternative to the primary use of G-CSF in all patients, especially in the palliative setting [15].

In a metastatic setting, to reduce clinical visits a lot of authors assumed might be appropriate to modify or delay the ICIs schedule (using 4-weekly nivolumab 480 mg or 6-weekly pembrolizumab 400 mg) [12]. Moreover, delaying the next cycle, missing certain cycles, or generally expanding intervals should be explored for patients who have been on ICI for more than 12/18 months. The possibility of stopping ICIs after two years should be considered, given the paucity of prospective information on the best treatment length for lung cancer [11].

Again, patients on chemotherapy could be discontinued treatment to minimize treatment-related immunosuppression or the risk of drug interaction [18]. In locally advanced NSCLC should be considered using durvalumab every 4 weeks instead of the standard 2 weeks.

Regarding radiotherapy, ESTRO-ASTRO underlined some recommendations, especially for lung cancer patients that are the highest-risk group. It could be preferable, when possible (such as in a palliative scenario), to consider hypo-fractionated radiation therapy respecting equivalent efficacy [16].

Nowadays, a new question mark is rising among oncologists: almost a year after the pandemic started, the first vaccines are authorized. It’s important to consider that anti-SARS-CoV-2 humoral and T-cell immune responses seem to be less effective in cancer patients, raising doubts regarding vaccine effectiveness [20]. On the other hand, not only cancer patients can be contagious and transfer SARS-CoV-2 for up to two months, but also they are at a considerably increased risk of morbidity and mortality; therefore cancer patients treated with systemic therapy should be considered a high–priority group for COVID-19 vaccination [21].

Thus, despite the few data concerning the vaccine’s safety and efficacy in cancer patients who had been or are being treated, guidelines recommended vaccination of all patients with cancer. On January 8, 2021, ESMO issued a call to action to the European Union Member States (EU MSs), highlighting the necessity of vaccinating all cancer patients as soon as feasible, particularly those on active anticancer treatment [22]. Recognizing the absence of evidence on immunocompromised patients’ ability to produce and maintain an immune response in pivotal trials [23], ESMO emphasizes the need of monitoring vaccine effects through appropriate studies and registries [24].

Certainly, more studies with longer follow-up periods are required to thoroughly examine the advantages and risks of COVID-19 vaccinations.

## Discussion

COVID-19 has represented a global emergency that forced us to quickly readjust the management of lung cancer patients to a new context. During the COVID-19 pandemic, oncologists were at crossroads. They had to face increased mortality of patients affected by COVID-19 and an excess of mortality for cancer patients, whose treatments had been de-intensified, delayed, or canceled [11]; therefore, physicians questioned how to guarantee the best care to the most fragile patients. An interesting paper by Pinato et al. [25] retrospectively evaluated 2,795 cancer patients diagnosed with SARS-CoV-2 reporting sequelae in 15% of cases, especially men, smokers, patients with comorbidities, and/or aged 65 years or older; sequelae were associated with an increased risk of death. However, the most interesting data was that, among patients on systemic anti-cancer therapy, 38.2% resumed treatment while 15% permanently discontinued therapy due to COVID-19 infection but only permanent treatment discontinuations were associated with an increased risk of death.

The recommendations extracted from literature are often based on expert opinion (mainly produced through the Delphi method) because they were generated in a short time due to the rapid advance of the pandemic. All guidelines and recommendations highlighted the relevance of being able to delay, if possible and based on risk stratification, any curative interventions (chemotherapy, radiation, surgery) [26]. Unfortunately, epidemiological data suggest that the pandemic seems not to be a conclusion time and could continue for months [25].

Due to the pandemic’s unclear duration, physicians had to evaluate the risk/benefit ratio of anti-cancer therapeutic strategy to do the best for the patients and to protect patients themselves, but also healthcare workers [9, 18, 27]. Therefore, the selected recommendations should be considered adaptable and flexible because they might be contextualized based on SARS-CoV-2 infection prevalence and the availability of diagnostic-therapeutic resources. It remains of fundamental importance to discuss each diagnostic and therapeutic decision with the patient taking into account risks and benefits that might vary from case to case.

## Abbreviations

ASTRO: American Society for Radiation Oncology
COVID-19: coronavirus disease 2019
CT: computed tomography
ESMO: European Society for Medical Oncology
ESTRO: European Society for Radiotherapy and Oncology
G-CSF: granulocyte-colony stimulating factor
GGO: ground-glass opacity
ICIs: immune checkpoint inhibitors
LCS: lung cancer screening
NENs: neuroendocrine neoplasms
NSCLC: non-small cell lung cancer
OS: overall survival
PCI: prophylactic cranial irradiation
PET: positron emission tomography
PORT: post-operative radiotherapy
PRRT: peptide radionuclide receptor therapy
SARS-CoV-2: severe acute respiratory syndrome coronavirus 2
SBRT: stereotactic body radiation therapy
SCLC: small cell lung cancer
SSAs: somatostatin analogues
TKI: tyrosine kinase inhibitors

## Declarations

### Copyright

© The Author(s) 2022.
